# Interface Behavior and Interface Tensile Strength of a Hardened Concrete Mixture with a Coarse Aggregate for Additive Manufacturing

**DOI:** 10.3390/ma13225147

**Published:** 2020-11-15

**Authors:** Arnošt Vespalec, Josef Novák, Alena Kohoutková, Petr Vosynek, Jan Podroužek, David Škaroupka, Tomáš Zikmund, Josef Kaiser, David Paloušek

**Affiliations:** 1 Faculty of Mechanical Engineering, Institute of Machine and Industrial Design, Brno University of Technology, Technická 2896/2, 616 69 Brno, Czech Republic; Vosynek@vutbr.cz (P.V.); Skaroupka@fme.vutbr.cz (D.Š.); Palousek@fme.vutbr.cz (D.P.); 2 Faculty of Civil Engineering, Department of Masonry Structures, Czech Technical University in Prague, Thákurova 2077/7, 166 29 Prague, Czech Republic; Josef.Novak.1@fsv.cvut.cz (J.N.); Akohout@fsv.cvut.cz (A.K.); 3 Faculty of Civil Engineering, Institute of Computer Aided Engineering and Computer Science, Brno University of Technology, Veveří 331/95, 602 00 Brno, Czech Republic; Podrouzek.J@fce.vutbr.cz; 4 Faculty of Mechanical Engineering, Institute of Physical Engineering, Brno University of Technology, Technická 2896/2, 616 69 Brno, Czech Republic; Tomas.Zikmund@ceitec.vutbr.cz (T.Z.); josef.kaiser@ceitec.vutbr.cz (J.K.)

**Keywords:** 3DCP, cementitious composite, concrete testing, concrete deposition, interface behavior, digital concrete, extrusion manufacturing, computed tomography, construction-scale 3D printing, large-scale additive manufacturing, Portland cement concrete, extrusion-based concrete print

## Abstract

3D concrete printing technology (3DCP) is a relatively new technology that was first established in the 1990s. The main weakness of the technology is the interface strength between the extruded layers, which are deposited at different time intervals. Consequently, the interface strength is assumed to vary in relation to the time of concrete casting. The proposed experimental study investigated the behavior of a hardened concrete mixture containing coarse aggregates that were up to 8 mm in size, which is rather unusual for 3DCP technology. The resulting direct tensile strength at the layer interface was investigated for various time intervals of deposition from the initial mixing of concrete components. To better understand the material behavior at the layer interface area, computed tomography (CT) scanning was conducted, where the volumetric and area analysis enabled validation of the pore size and count distribution in accordance with the layer deposition process. The analyzed CT data related the macroscopic anisotropy and the resulting crack pattern to the temporal and spatial variability that is inherent to the additive manufacturing process at construction scales while providing additional insights into the porosity formation during the extrusion of the cementitious composite. The observed results contribute to previous investigations in this field by demonstrating the causal relationships, namely, how the interface strength development is determined by time, deposition process, and pore size distribution. Moreover, in regard to the printability of the proposed coarse aggregate mixture, the specific time interval is presented and its interplay with interface roughness and porosity is discussed.

## 1. Introduction

Digitalization in the construction industry could be perceived as being at an early birth stage, despite earlier attempts to develop robotic construction systems [1].

The correlation between productivity growth and the digitalization index is well known; therefore, it is no surprise that the construction sector has a lower digital index, with productivity growth localization being in negative values. Currently, for example, one-third of the world’s energy consumption and CO_2_ emissions production is due to the construction industry. The main aim of additive manufacturing (AM) is to quantitatively minimize this impact on the environment and loss of life on the construction site [2].

AM used in the construction industry, called 3D concrete printing technology (3DCP), has developed rapidly in recent years. While being more experimental than general, it is already used for the construction of small-sized buildings, complex-shaped prefabricated concrete elements, and constructions without requiring the use of expensive formwork. However, this technology has several issues that have not yet been fully resolved, though autonomous construction systems have the potential to improve the speed, quality, and safety of the onsite fabrication of architectural-scale structures under various environmental conditions [3,4].

As the principle of 3DCP technology consists of concrete layering over time intervals, one of the major issues is the interface strength between adjacent layers. However, it is important to point out that once a very short time interval passes, the bearing capacity of an extruded concrete layer is not high enough to withstand the dead load resulting from layers cast on top of it. Moreover, the adhesion quality worsens with the increasing length of the print-time interval [5]. Consequently, both of these aspects should be carefully considered when print-time intervals are proposed. According to Model Code 2010, there are parameters that influence adhesive bonding, such as roughening of the interface, the strength class of old and new concrete, the quality of the top layer of old concrete, the porosity and moisture content of the old concrete, and the quality properties [6].

There are a few experimental studies that have dealt with the effects of the print-time interval on the adhesion strength of 3D-printed concrete layers. One of these studies [7] focused on the interface strength between 3D-printed concrete layers in print-time intervals ranging from 15 min to 7 days. The findings obtained from direct tension tests demonstrated that the adhesion strength decreases with increasing print-time intervals. Another study [8] dealt with the effect of surface moisture on adhesion strength. The obtained experimental results showed that the relation between the surface moisture and adhesion strength is very complex. Moreover, the adhesion strength is significantly affected by many aspects, such as the printing process, evaporation rate, and bleeding rate of concrete mixtures. The main conclusion of the study was that a dry surface did not have the right conditions for bond development [5].

Using a different approach, Rubio et al. demonstrated that printed mortars exhibited improved mechanical behavior compared with cast specimens. This was due to extrusion, which has a positive effect on the density of the mortar because of the fast layering process that hides defects, though this process does not enable the creation of cold joints [9]. Note that Nerella et al. provided evidence that good adhesion quality at the layer interface displays quasi-isotropic behavior, resulting in a better connection than for cast specimens [10].

Both studies investigated the interface strength between extruded concrete layers with respect to different boundary conditions. As the experiments were conducted on cement-based composites with only fine aggregates, the presented experimental investigation was proposed with the aim of observing the interface strength of concrete with coarse aggregates in the form of crushed stone with a maximum nominal size of 8 mm, which is used only rarely at present for 3D concrete printing technology [11,12]. Based on existing knowledge, it is necessary to highlight that measurement of the interface strength in this field is still an open question, and it is one of the key factors for our motivation to work in this field, where this study attempted to provide complementary data.

## 2. Materials and Methods

Portland cement concrete that was suitable as material available for use in AM and formed using 3DCP was manufactured during this project. The key mechanical requirement of a cement-based composite was a high strength that is sufficient to withstand compressive stress during early formation (grades of cement as per European Standard–EN-197-1).

The proposed concrete composition was as follows (mass per one cubic meter):

- 400 kg Portland cement (OPC) conforming to the European standard, strength class 42.5 R;
- 1130 kg fine aggregate 0–4 mm;
- 300 kg coarse aggregate—crushed stone 4–8 mm;
- 100 kg Metakaolin, Mefisto L05, České Lupovské závody, Pecínov, CR, Czechia;
- 3 kg liquid solidification accelerator, Betodur A1, Stachema, Kolín, CR, Czechia;
- 285 kg of water.

Cement-based composites for 3D printing technology should be adapted mainly to meet requirements for fresh concrete, such as in terms of extrudability and buildability. These properties are mutually related to the workability and open time of a fresh mixture. Prior to the concrete production, the relative humidity and temperature of each concrete component were measured (Table 1).

The initial setting time of the cement paste consisting of cement, water, liquid solidification accelerator, and Metakaolin was intended to define a time frame in which it was possible to print using the concrete mixture. However, as the Vicat apparatus test was not developed particularly for 3D concrete printing, an extrusion-based test focused on the quality control of the deposited concrete layers was also performed.

The components of the mixture (fine aggregate, coarse aggregate, Portland cement, Metakaolin) were dry-mixed for about 2 min. Then, tap water with liquid solidification accelerator Stachema Betodur A5 was added immediately, and mixing was continued for about 2 min. After mixing, the mixture was homogenous and formed into a consistent fresh state (visually assessed). Subsequently, a concrete slump test was undertaken to observe the workability of the proposed fresh mixture.

**Figure 1:**
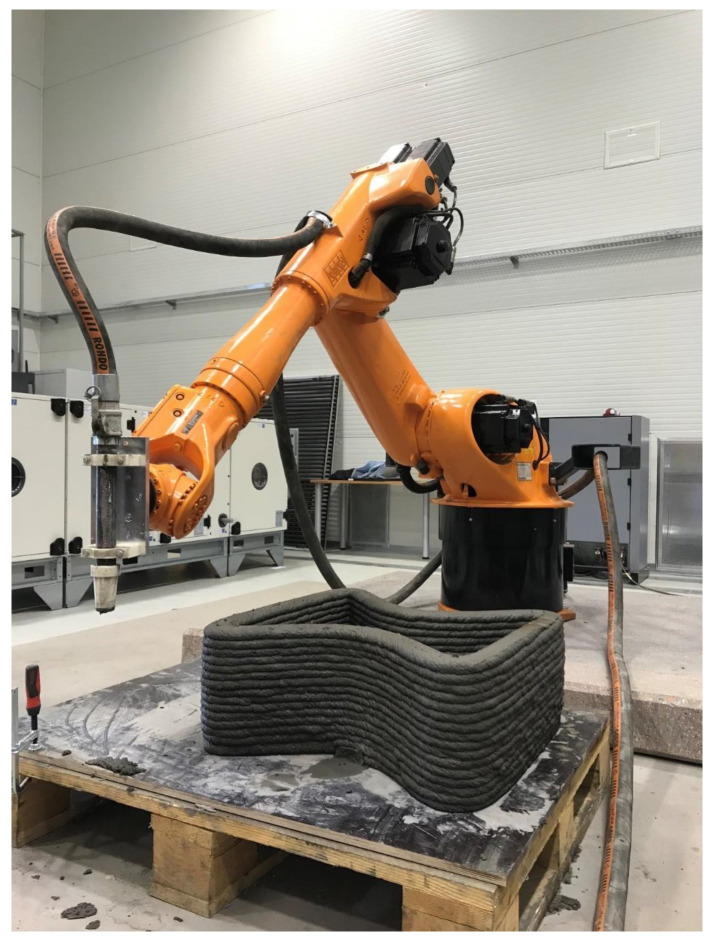
3D printing of a geometric element.

The main objective of the extrusion-based test was to print single concrete layers at various elapsed times from the initial mixing of the concrete components and to subsequently control the quality of the deposited layers visually. The layers were extruded using a specially designed extruder (Figure 1) at a constant speed of 10 mm/s in a horizontal direction. Then, the quality of each concrete layer was visually assessed.

Based on the obtained findings, the so-called open time was determined, which defines a time in which the fresh concrete is still workable enough for 3D printing technology. The best quality of extrusion was obtained in the open time from 20 to 40 min. Once the open time was determined, 20 concrete specimens were prepared from 4 fabricated specimens for the uniaxial tension test. First, the concrete mixture was deposited into the form mid-height (25 mm) 20 min after the initial mixing of concrete components. Second, the other layers were deposited at five-minute intervals into the 50-mm-high form, and the formwork was then covered with cling film in order to avoid moisture loss. After 28 days, the whole concrete elements were cut into 24 concrete specimens. Twenty specimens were bonded to steel plates using epoxy glue to measure the interface strength, and 4 specimens were used for computed tomography (CT) analysis to analyze the porosity and distribution of the coarse aggregate.

All parameters were retained for repeatability with print tracks using 3DCP.

### 2.1. Setting Time and Workability—The Vicat Apparatus Test

The Vicat apparatus test was performed on the cement paste with the aim of observing the setting time of the proposed mixture to determine for how long the concrete mixture might be workable enough for 3DCP. First, it was necessary to find the adequate content of mixing water required to produce the normal consistency of a cement-based mixture, which was subsequently used for determining the setting time in accordance with EN 196-3. The mixture was composed of cement and Metakaolin was used as a partial cement replacement. Subsequently, the mixture was used for observing the initial setting time and final setting time in accordance with the same standard [8].

### 2.2. Extrudability—Extrusion-Based Test

The principle of assessing the extrudability involved setting the exact time interval after the mixing process in which to print single layers. Time intervals of 5 min were used, followed by subsequent visual observation to assess the print quality. Note that the same method was used in a study by Ma et al., where the findings show that with good extrusion of the mixture, violation of the continuity of the printed trace in the form of cracks was eliminated [11].

### 2.3. Deposition into the Form

**Figure 2:**
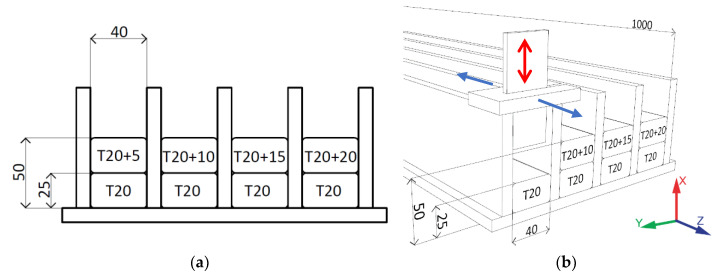
(**a**) Schema represents the layer deposition time intervals, dimension units in millimeters; (**b**) schema of the forms and the adjustable trowel, dimension units in millimeters.

A unique method for the manufacture of concrete elements was developed that was similar to the formwork method. The form for the concrete specimens and trowel with adjustable heights of 25 and 50 mm were built. Each layer was deposited into the cast and wiped off in the positive *Z*-axis direction using a trowel to demonstrate the sliding of the nozzle during the 3DCP process (Figure 2b). The form dimensions and troweling height were chosen according to the nozzle cross-section to be used in 3DCP. The parameters (T5, T10, T15, T20) indicate the time interval between depositions (Figure 2a,b). The 4 elements were manufactured in a form of 1 m long bars, which were cut into 24 specimens.

### 2.4. Scanning of the Specimens

**Figure 3:**
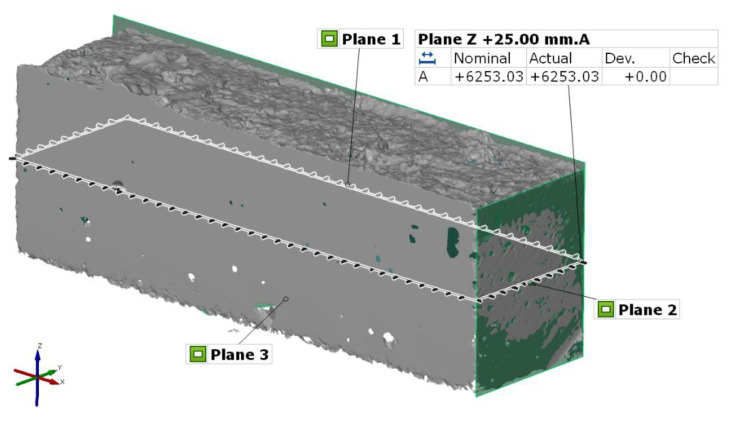
Cross-sectional area of a specimen measured in GOM Inspect (GOM) analytical software.

Twenty specimens were scanned using the 3D optical scanner Atos TripleScan 8M (GOM GmbH, Braunschweig, Germany). This scanning apparatus was used for assessing the real area of the layer interface to determine the exact dimensions. Analytical software GOM Inspect 2018 (GOM GmbH, Braunschweig, Germany) was used to obtain more accurate specimen dimensions, especially for the real area of the layer interface (Figure 3). The principle of this scanner is based on active fringe projection. The measurement of objects of various sizes was made possible by changing the lenses of the CCD (Charge-Coupled Device) cameras and the projector, in addition to the adjustable angle between the cameras. A programmable rotary table was used for the measurements, and the parameters of the 3D scanner were set according to VDI/VDE 2634, Part 3: “Optical 3D-measuring systems, multiple view systems based on area scanning” (Table 2) [13].

### 2.5. Interface Strength

Uniaxial tests were conducted to measure the interface strength. Note that which testing method to use to determine the interface strength of the printed concrete is still an open question.

It was necessary to cut four initial precast concrete elements with dimensions of 50 × 40 × 1250 mm^3^ into twenty specimens with dimensions of 50 × 40 × 150 mm^3^. The cross-section of concrete blocks (40 × 50 mm^2^) was selected to simulate two concrete layers produced using 3D concrete printing technology with nozzle dimensions of 25 × 40 mm^2^. Subsequently, steel T-profiles were glued to the top and bottom of each block with the epoxy glue Sikadur-31 EF (Sika AG, Baar, Switzerland) with a tensile strength of 20 N/mm^2^ after 3 days of drying at a temperature of +23 °C [14].

Attention was paid to accurately align the specimens in the machine to obtain pure tension and avoid eccentricity that would distort the results.

The uniaxial tension tests were performed on the prepared specimens with the aim of observing how the interface strength varied with respect to the time interval between the printing of two adjacent concrete layers.

The tests were conducted with a displacement rate of 1 mm/min in a Zwick Materialprufüng Z020 device (ZwickRoell Group, Ulm, Germany)with a 20 kN load cell [15]. The obtained data were used for the calculation of the peak interface strength using engineering stress Equation (1), where *F* and *A* are the applied tensile force and nominal cross-section area of the specimens, respectively:

*σ* = *F_n_*/*A*. (1)

### 2.6. CT Scan Analysis of Specimens

**Figure 4:**
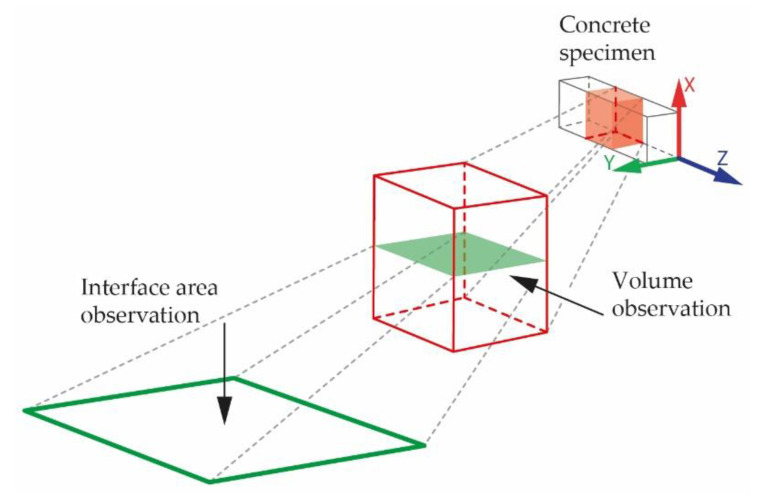
Direction of observation in the volumetric analysis and interface area analysis.

The last four specimens were visualized using computed tomography (µCT, GE phoenix v|tome|x L240). The main parameters of the X-ray tube were a voltage of 180 kV, current of 200 µA, and a 0.5 mm thin copper plate filter. The measurement was focused only on the selected region (Figure 4), which allowed for a 38 µm linear voxel resolution. The CT reconstruction (using the backprojection algorithm) was performed in the Datos reconstruction software (GE Sensing & Inspection Technologies GmbH, Wunstorf, Germany). All subsequent post-processing was performed in the software VGStudio MAX 3.3 (Volume Graphic GmbH, Heidelberg, Germany).

The software analyses aimed to determine the porosity ratio and distribution of the cement-based composite. Both ratios were calculated using a thresholding algorithm for determining the boundary between the materials. The threshold used for porosity detection was determined automatically by the software according to peaks of the data histogram representing the air (background) and other materials for better comparability of the results. Only the closed pores (surrounded by material) were considered in the analysis results. Since the histogram did not allow one to clearly identify different materials in the specimen, the threshold used for white cement-based composite detection was selected by the operator individually for each specimen according to visual inspection in representative cross-sections. Because the cement-based composite was not completely homogenous, the results reached by the thresholding approach were optimized using the erode/dilate function. Cement-based composites were captured in the data as regions of interest. Their distribution was then expressed as a proportion of the region volume to the whole specimen volume.

## 3. Results and Discussion

### 3.1. Hypotheses—Theorem

The main objectives of the study were to analyze the tensile strength at the interface of the 3D-printed concrete layers and to validate three main hypotheses formulated in the scientific literature:

1. The interface force should have the same trend as described in the study by Le et al. [16] and Sanjayan et al. [8], where the strongest adhesion force would be obtained up to 15 min after the layer deposition for cementitious materials.
2. The interface strength is affected by the number of pores and their sizes, in addition to interface roughness, which depends on the aggregate size and type of material used. Concrete elements that are 3D-printed out of aggregate particles are supposed to fail at the layer interface when the strength of the cement paste matrix is exceeded.
3. During the process of the deposition of a material with a higher fraction of coarse aggregate, there is a probability that the roughness of the first-layer surface could cause air bubble locking due to the deposition of a second layer. The assumption is that with a large time gap, a large number of pores would be observed at the interface area close to the coarse aggregate grains.

### 3.2. Setting Time and Workability

#### 3.2.1. Setting Time and Workability—The Vicat Apparatus Test

**Figure 5:**
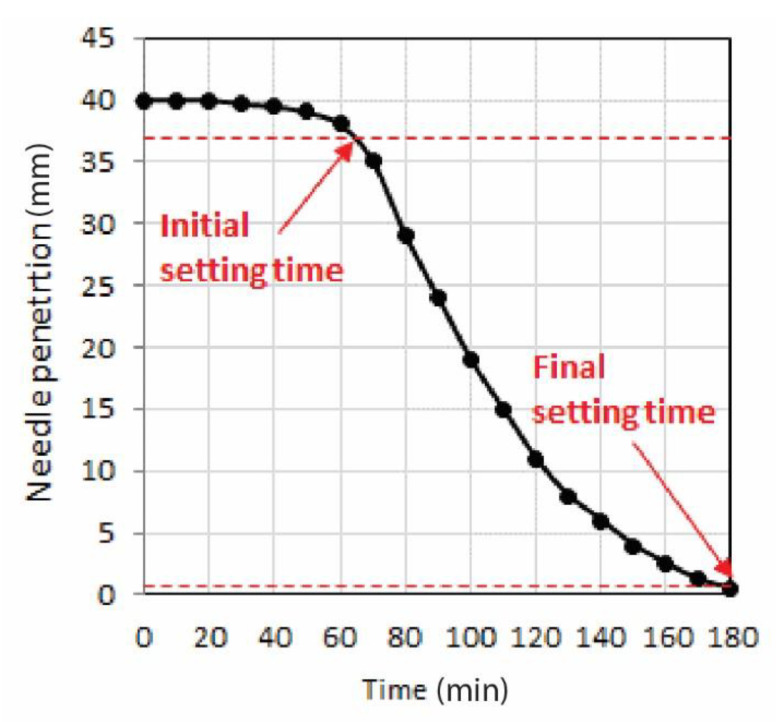
Vicat test of the cement paste consisting of cement, liquid solidification accelerator, Metakaolin, and water.

The experimental data obtained from the Vicat test were used for the elaboration of the graph presented in Figure 4, which shows the development of the cement paste with the solidification accelerator Stachema Betodur A5 setting time. The initial setting time was found to be 65 min from the initial mixing of all components. This time fulfills the requirements for cement class 42.5 in accordance with ČSN EN 196-1 ed.2. As the initial setting time can be defined as the time when the cement paste starts losing its plasticity, the concrete mixture was assumed to have sufficient workability for 3D concrete printing until 65 min from the initial mixing of the concrete components (Figure 5).

The operating assumption of the Vicat apparatus test was verified using the extrusion-based test when investigating the extrudability of the mixture, where the gathered data show a partial relation between the results from the Vicat apparatus test and the extrusion-based test’s optimal open time.

#### 3.2.2. Extrudability—Extrusion-Based Test

**Figure 6:**
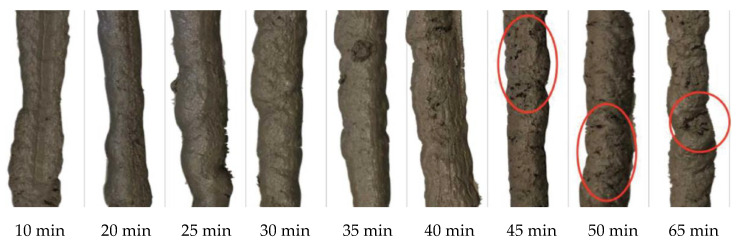
Quality of the single-layer extrusion: observation of the fluid consistency of extrusion from 10 min after mixing and crack occurrences from 45 min after mixing.

The principle of the test was to print single layers in time interval spans of 5 min ranging from 10 to 65 min, as counted from after the mixing was finished, and to subsequently visually observe the layer quality. The obtained findings demonstrate that the layer extruded 10 min from the initial mixing of components was not able to retain its shape due to the very fluid consistency of the concrete. In contrast, the layers extruded later than 40 min from the initial mixing of components showed shape discontinuity and minor cracks that likely resulted from the concrete consistency being too viscous. Consequently, the optimal print time of the proposed concrete mixture was between 20 and 40 min from the initial mixing of concrete components (Figure 6). Note that regarding extrudability, the gathered data showed a partial relation between the results from the Vicat apparatus test and the extrusion-based test’s optimal open time.

While the number of cracks in the extruded layers was not affected by the age of the cementitious composite too much, the width of the cracks rapidly increased with increasing time. In particular, the crack width was equal to 0.3 mm and nearly 1 mm at the time 45 min and 65 min, respectively. However, it is important to point out that only a visual check was conducted and a deep investigation of this phenomenon should be carried out in the future.

### 3.3. Deposition into the Form

**Figure 7:**
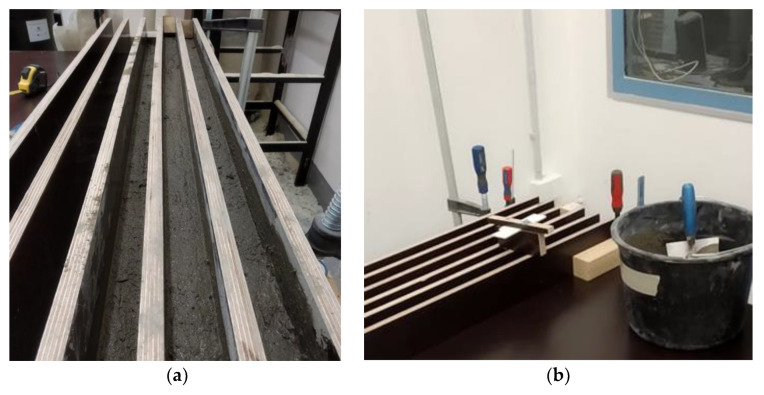
Forming process. (**a**) Forms with deposited concrete, (**b**) Forms with adjustable trowel.

For the deposition of concrete, the forms and trowel with adjustable heights were built (Figure 7). The deposition of each first layer was done 20 min after wet mixing (denoted as T20) and trowelled at a height of 25 mm. The deposition of the second layer was conducted at a 5 min interval after 20 min of wet mixing (Figure 2a) and troweled at a height of 50 mm. The troweling of layers was equal to the nozzle movement with a velocity of approximately 10 mm/s for the 3DCP process. After the layer deposition, the concrete elements in the forms were covered by wet textile and plastic foil to preserve the microclimatic conditions at an ambient temperature (23.7 ± 2 °C).

### 3.4. Scanning of Specimens

Each specimen was optically scanned after being cut off from the concrete element. The optical properties (surface matt and reflection) meant that the specimens were suitable for direct scanning, where coating of the surface with TiO_2_ was not required [13]. The boundary of deposited layers was measured from the bottom of the concrete elements. The three planes were fitted onto the bottom, left, and right side of the specimen using the Gaussian best fit method with the use of Three-sigma rule (i.e., three standard deviations). After that, the specimen was aligned into the local coordinate system. The Z-axis coordinate of the interface area (Figure 3) was set as 25 mm from the bottom plane, and to define the exact area, the single-section function was used. After this, the cross-sectional area was measured by the area analysis function (Table 3).

### 3.5. Interface Strength

The interface strength was based on the equation of engineering stress (Equation (1)). The results from the proposed experiment demonstrated the assumption about the relation between the adhesion strength and time interval between the deposition of concrete layers. It was evident that the interface tensile strength gradually decreased from 2.6 to 2.1 MPa as the time interval increased. This investigation also showed that the time interval between the deposition of concrete layers affected the failure mode of the test specimens.

The probability of specimen failure in the interface area was the most significant when using a delay time of 20 min with a 100% failure rate, then 15 min with an 80% failure rate, 10 min with a 40% failure rate, and 5 min with a 20% failure rate.

**Figure 8:**
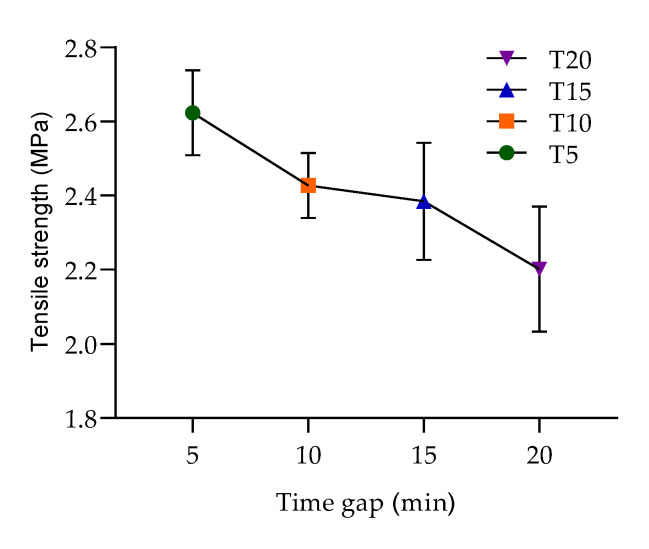
Interface failure trend of the specimens with different time depositions of the bottom layer, which are given in more detail in the descriptive statistics (Table 4).

The values show that the interface strength of the deposited layers was significantly influenced by the delay time used for the layer deposition. The interface strength was strong at 5 min, and from this delay time, there was a decreasing trend between specimens deposited at 10 and 15 min, where a small fluctuation was measured (Figure 8). A similar trend can be observed in the study by Panda et al. [17]. This also partially matched the observations of another study, where the authors proclaimed that, generally, regarding printing speed, the interface strength decreases when the time gap between two layers increases, and the interface strength is stronger in specimens printed at a lower speed [18]. In this study, a constant speed was used for all mixture deposition processes with different time delays. This helped to avoid undesirable roughness manipulation of the upper surface of a deposited layer due to the speed of the nozzle. This process avoided the deformation of the printed layers due to the method used for deposition into the form. This method enabled reaching a more accurate result with a lower variance in the data for this specific mixture, and its was more likely that a similar trend would be observed for 3D-printed specimens. Note that Rubio et al. found that heterogeneity was induced by 3D printing through extrusion/deposition based on the density of mortars. The density of the upper layers was lower than that of the bottom layers after the deposition/printing. This means that lower layers began to consolidate. This phenomenon of consolidation has a direct impact on the resistance levels [9] and requires more comparative investigations to characterize this impact.

**Table 4:** Descriptive statistics.

Specimen	T5	T10	T15	T20
Number of values	5	5	5	5
Minimum (MPa)	2.492	2.294	2.162	2.013
Maximum (MPa)	2.745	2.520	2.591	2.457
Range	0.253	0.227	0.429	0.444
Mean (MPa)	2.623	2.427	2.384	2.201
Std. deviation	0.114	0.088	0.158	0.169
Std. error of the mean	0.051	0.039	0.071	0.076
Coefficient of variation (%)	4.358	3.608	6.624	7.668

**Figure 9:**
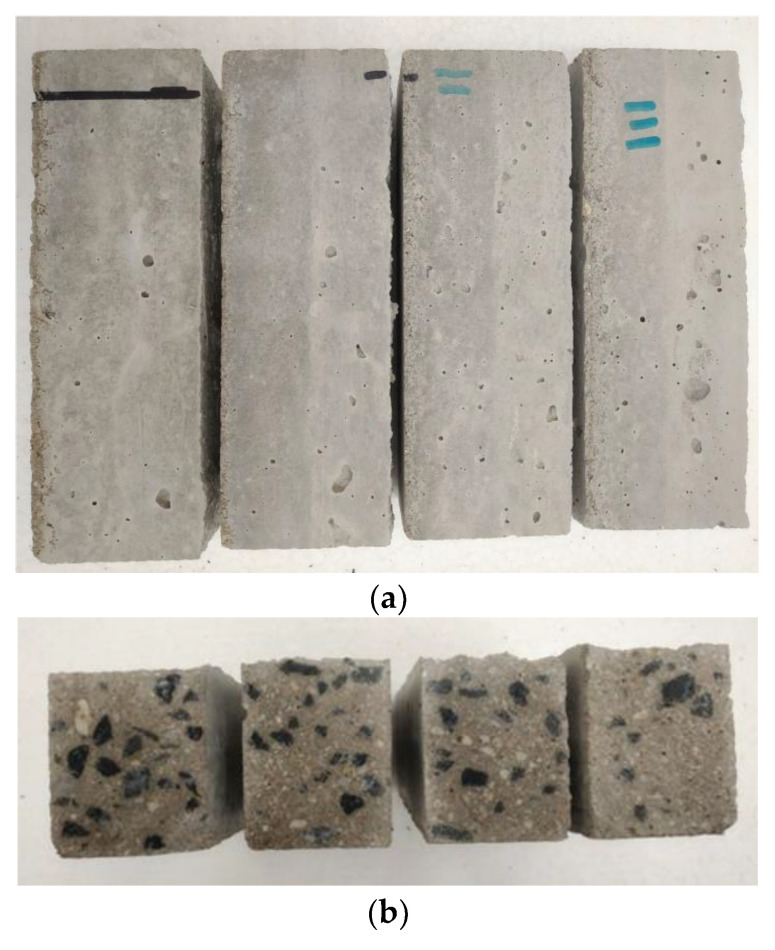
Section view of specimens and details of the layer boundaries (5, 10, 15, and 20 min from left to right). (**a**) side view—visible boundaries; (**b**) section view.

**Figure 10:**
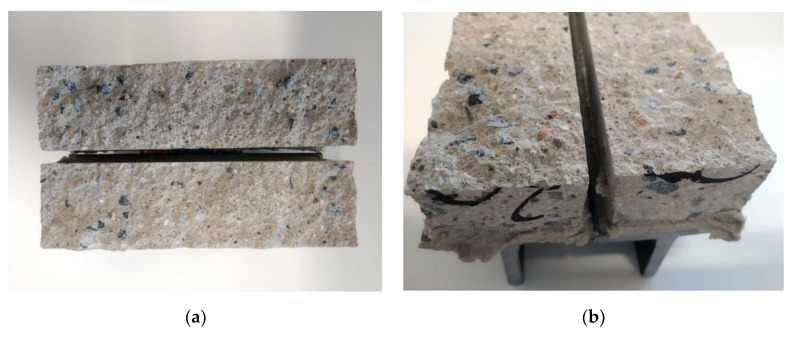
Failure of the specimens on the layer interface. (**a**) upper view; (**b**) detail view.

The failure area of all the specimens was not smooth, as is typical in fine-grained concrete mixtures [8]. The cracked areas had rough surfaces, and the visible coarse aggregate particles with sizes of 4–8 mm were cracked. This means that the coarse aggregates penetrated into the bottom layer, which is evident in cross-sectional areas (Figure 9 and Figure 10a,b).

The interface area, where the old and new concrete mixtures interact, can be considered as a thin layer consisting of very fine particles. This thin film is usually localized on the outer surface of the fresh mixture and functions as a lubricant between the mixture and pipe during the pumping process. The results in this study confirmed the explanation of the results from the study of Panda et al., where they explained the differences between the roughness on interface surfaces that were deposited/3D-printed at different times. The roughness decreased with the time delay interval, which correlated with the drying of the thin-film layer. This means that the thin-film layer diminished and the adhesion of the layers was no longer efficient [17]. The authors found a correlation between the time delay interval and the moisture effect phenomenon and entrapped air at the interface area, which can cause low interface strength [17]. This behavior was clearly observed in the specimen CT scans.

**Figure 11:**
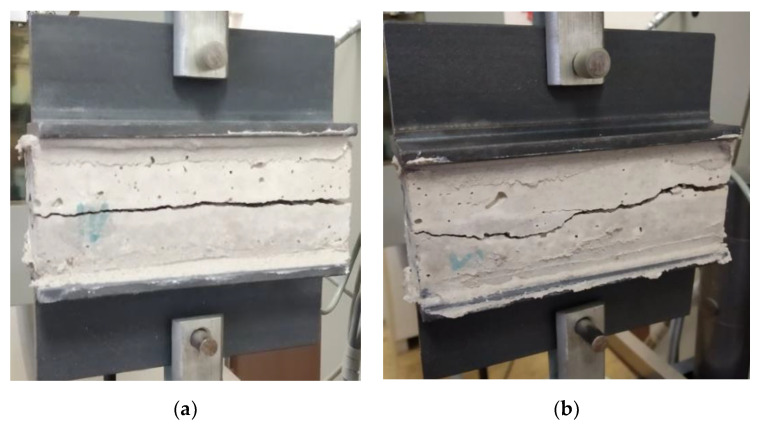
Specimen crack patterns: (**a**) regular failure on the layer interface areas and (**b**) an irregular crack.

In the direct tensile test of the specimens, two kinds of crack patterns were observed: the first kind of pattern (Figure 11a) was characterized by a crack that spread through a single surface, while the second kind of pattern was characterized by a crack that spread through multiple surfaces (Figure 11b). This means that for the first kind of crack pattern, a lower amount of energy was needed compared to the second kind of pattern. Multiple cracks could be caused by a secondary flexure due to a crack arrest by 4–8 mm coarse aggregates or due to local tension softening in the weakest part of the specimen [19]. Note that even though the specimens were previously bonded to metal anchors and aligned into the device, shear stress may have occurred due to an interlocking mechanism [6,20].

### 3.6. Porosity

The geometric shape of the pores and their size and distribution within the whole volume directly affects the concrete strength and its durability due to their influence on the action of forces. The pore structures inside a concrete mass are very complex and have variable size scale distributions. The size of pores ranges from the nanoscale to millimeter size, while the majority of pores are formed as a result of the mixing process and evaporation of excess water [21,22].

The size of pores can be sorted into three basic categories according to their relative diameters:

- 0.5–10 nm gel pores
- 10 nm–10 µm capillary pores
- 50 µm–1 mm air pores.

As seen in the specimen failure detail (Figure 10a,b), high porosity was observed in the failure areas. During the layer deposition process, a higher concentration of air pores in interface areas arose, which provided a higher occurrence of stress concentrators [16,18]. As indicated in previous studies, the pore size has a significantly negative effect on the interface strength of deposited layers [16].

#### 3.6.1. Volumetric Analysis

**Figure 12:**
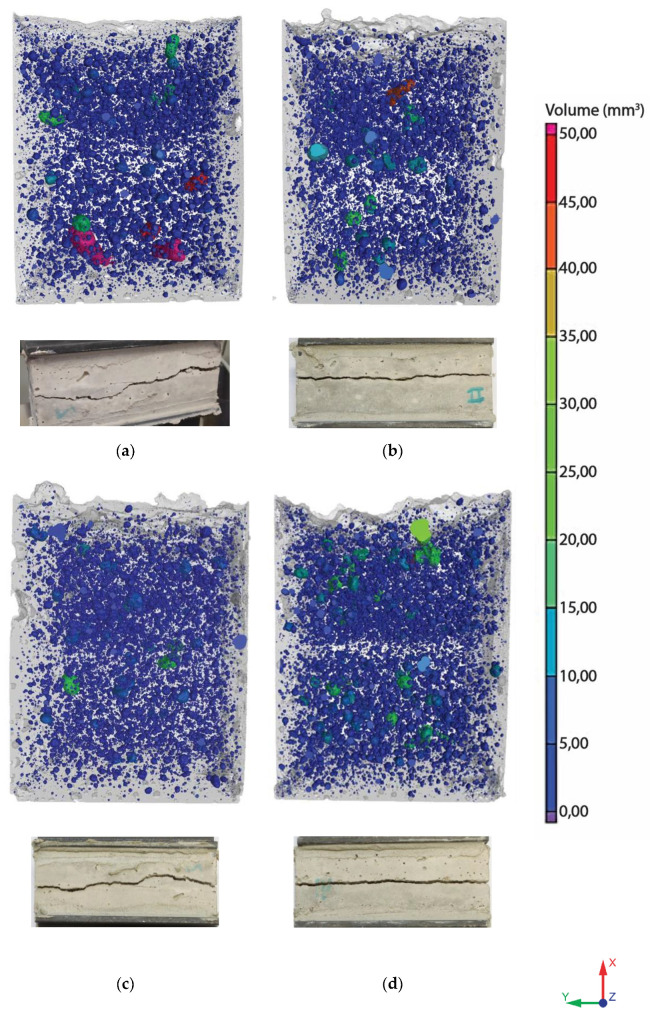
Comparison of the pore distribution and sizes for different entrapping times, illustrating the common crack patterns of the specimens. The computed tomography (CT) porosity of the specimens at different layering times, where the parameters are detailed in Table 2: (**a**) specimen T5, (**b**) specimen T10, (**c**) specimen T15, and (**d**) specimen T20.

For the CT, the resolution of 38 µm was set according to the size of the specimens and the relevant size of pores based on previous studies. The size recognition of pores is dependent on the resolution and went from 50 µm to several millimeters. The volume analysis showed that the defect volume ratio had different results for each specimen (Table 5), but with similar characteristics. Figure 12 shows a declining number of closed pores of a specific size, which was correlated with the stage of hardening and the deposition time.

The bottom layer of the T20 specimen was hardened enough and much less affected by the compressive force from the upper layer. This allowed us to observe the effect of moisture inside each layer structure. Figure 12 shows the migration of a large number of pores with different sizes in each specimen. This was explained by the moisture exchange phenomenon, where concrete from the bottom layer was drier and absorbed moisture from the upper layer [17]. Due to this process, small air bubbles from the bottom layer escaped vertically. As a consequence of this movement, small air bubbles coalesced together, resulting in the presence of large pores in the bottom layer and small pores in the upper layer. The presence of large air bubbles at the center could be explained as captured air that caused roughness on the surface of the first layer [15,16].

**Figure 13:**
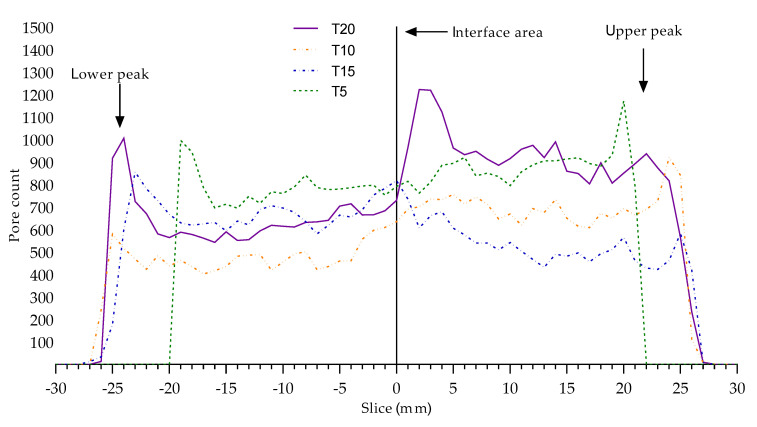
Qualitatively different pore distribution trends related to the moisture exchange phenomena; note, e.g., the symmetric peak locations of T5 vs. T20.

**Figure 14:**
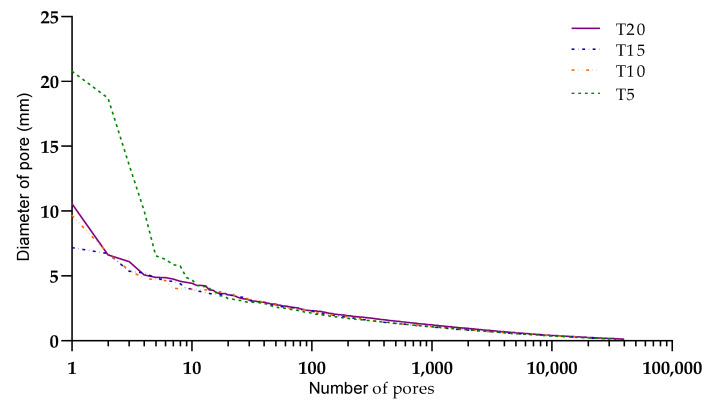
Volumetric pore distributions based on volumetric CT scans, which are described in more detail in Table 5.

Figure 13 shows the pore count in the Z height coordinate (X-axis on the graph). The height of the specimens was the same and the onset of pores differed due to the freshness of the cement mixture and the water content. The pore migration in the fresh mixture was faster than in older mixtures. This difference is evident in specimen T5. The layer interface area had significant boundaries, where we observed a trend of a rapid increase in pore count. The specimen T15 has an opposite trend, which could be related to a moisture exchange phenomena due to the mixing procedure. This anomaly requires further in-depth investigation. For better understanding the Figure 14 shows volumetric air pore distribution of the specimens related with the Table 5.

#### 3.6.2. Area Analysis

**Figure 15:**
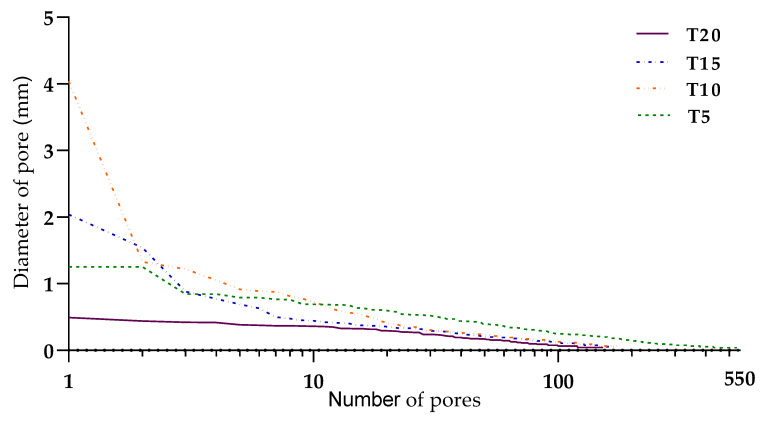
Pore distributions at the interface area based on cross-sectional CT scans, which can be found in more detail in Table 6.

**Figure 16:**
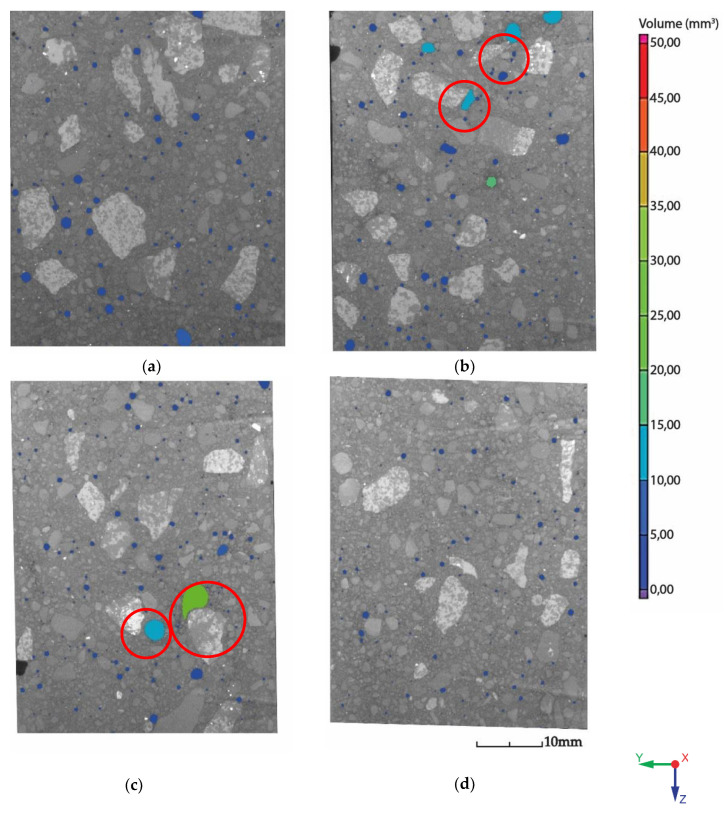
The CT porosity cross-section at the layer interface area, which can be found in more detail in Table 6, and localized large air bubbles entrapped near the coarse aggregate grains: (**a**) T5, (**b**) T10, (**c**) T15, and (**d**) T20.

The area analysis of the air pore distribution (Figure 15) provided a 2D view on the interface area (Figure 4 and Figure 16). The air pores that are shown (Figure 15) related with Table 6 were entrapped at the interface area of specimens T5–T20 in the concrete mass (Figure 13). Large entrapped air bubbles or clusters of small voids near the coarse aggregate grains could be explained as a consequence of deposition and trowel processes, where the increased time of layer deposition can cause a higher abundance of bigger pores [18]. The interface area was included to compare the results obtained from the volumetric analysis in order to show which was more significant.

## 4. Conclusions

In summary, this experimental study was proposed with the aim of observing the influence of the time interval between casting concrete layers on the tensile strength at their interface. Two-layer concrete elements with different layer deposition times were manufactured, with etalon used as a measure for the extrusion-based 3DCP. The mix composition was the same during all experiments. The results from the uniaxial tension test enabled us to draw the following conclusions. 

### 4.1. Hypotheses Validity

(1) The results supported the first hypothesis and the assumption about the development of interface strength in relation to the time interval between the casting of concrete layers. The interface strength decreased with an increasing time interval. This matched a study by Panda et al., where a similar trend was observed [17]. This also partially matched with one study where the authors proclaimed that, generally, regarding printing speed, the interface strength decreases when the time gap between two layers increases, and the overall strength is stronger in specimens printed at a lower speed [18]. In this study, the speed was constant in each deposition process of a mixture with a different time delay. This means that the process avoided undesirable roughness manipulation of an upper surface of a deposited layer due to the speed of the nozzle.
  In addition, Rubio et al. conducted a study in which printed mortars exhibited improved mechanical behavior compared with cast specimens. This was due to extrusion, which has a positive effect on the density of the mortar because of the fast layering process that hides defects, though this process does not enable the creation of cold joints. The study found that heterogeneity was induced by 3D printing through extrusion/deposition on dense mortars. The density of the upper layers was lower than that of the bottom layers after the deposition/printing. This means that lower layers were consolidated. This phenomenon of consolidation has a direct impact on resistance levels [9]. Note that Nerella et al. showed that the good adhesion quality at the layer interface has quasi-isotropic behavior, with better connection than a cast specimen [10].
(2) The geometric shape of the pores and their size and distribution within the whole volume directly affects the concrete strength and its durability by influencing the action of forces [22]. During the layer deposition process, a higher concentration of air pores arises on interface areas, which provides a higher occurrence of stress concentrators [16,18]. As indicated in previous studies, the pore size most likely has a negative effect on the interface strength of the deposited layers [16]. This study showed that during specimen failure, high porosity was observed at the failure areas, which was evidenced via volumetric and area analysis of the CT scans. However, in this causal study, the volumetric analysis showed pore size distribution and the exact position of pores inside specimens. It was shown that large air pores were outside this study’s area of interest: they did not conspicuously affect the interface area and they had a low impact on the interface strength data. The 4–8 mm sized coarse aggregate caused surface irregularities by penetrating into the next-deposited layer on the bottom, as demonstrated via CT scanning of the specimens. Note that interface roughness is one of the parameters that influence adhesive bonding [6] and the most likely improvement in adhesion strength due to the size of the coarse aggregates was not demonstrated to be correlated with the crack occurrence behavior. Multiple cracks could be caused by secondary flexure due to crack arrest by 4–8 mm coarse aggregates or local tension softening in the weakest part of the specimen [19]. Note that even though the specimens were previously bonded to metal anchors and aligned into the device, there was the possibility of shear stress occurring due to an interlocking mechanism [6,20]. However, this was not within the main scope of this study.
(3) Based on the first and second hypotheses concerning the deposition process, Van Der Putten et al. concluded that increasing the time delay to deposition induces a higher number of bigger pores in the lower and central parts of a specimen [18]. This was correlated with a fresh stage of deposited material. The CT area analysis showed a partial match with the literature and the hypothesis that the entrapped air bubbles were due to the deposition process near where the large coarse aggregate grains were localized was confirmed. However, the relation between the time gap and the increased occurrence of large air bubbles was not demonstrated.

### 4.2. General Findings

(1) Based on previous studies, the interface strength is related to the roughness of the surface, pore size, and thickness of a thin film of very fine particles [17]. The size of coarse aggregates does not conspicuously influence the uniaxial tension strength, but it may influence crack propagation. Note that even though the specimens were previously bonded to metal anchors and aligned into the device, there was the possibility of shear stress occurring due to an interlocking mechanism [6,20]; this can cause distortion of the interface strength.
(2) The propagation of cracks was proven to be related to the homogeneous arrangement of pores and aggregate (distribution and size) throughout the specimen, which was related to the effect of moisture and pore migration depending on the setting stage of the concrete.
(3) Regarding the test specimens in which the material was layered 20 min after mixing, the layers did not bond perfectly due to solidification. This resulted in inhomogeneity of the pores throughout the specimen and, thus, inhomogeneous overall hardness. In the direct tensile test, these specimens were broken exactly at the layer interface.
(4) The results obtained from the extrusion-based test performed within the scope of the work demonstrated that it was possible to print from the proposed mixture within 20 to 40 min of the initial mixing of concrete components, and the results obtained from uniaxial tension testing indicated that layer deposition was appropriate for 3DCP in terms of good adhesion between layers within a time interval from 5 to 10–15 min.
(5) In the case of 3DCP, the lower interface strength was predicted on the basis of the volume ratio and area ratio of porosity, and the stress concentration that occurred during the 3DCP process in the layer deposition. This determined the crack pattern and specific specimen failure [23].

It is necessary to highlight that the correlation between the time delay of deposition and interface strength was established for the specific defined material and specific surface conditions. This study established a basic etalon measure to determine the interlayer strength with 3DCP and formwork technology. It is necessary to investigate the influence of surface conditions of the contact surfaces between layers on the interface strength for 3DCP for every mixture.

## Figures and Tables

**Table 1:** Conditions of material components and ambient environment.

Symbol	Portland Cement (OPC)	Fine Aggregate	Coarse Aggregate	Metakaolin	Ambient Environment
RH (%)	42.18	95.33	52.13	40.62	63
T (°C)	23.15	20	22.46	23.11	23.7

**Table 2:** Parameters of the 3D scanner GOM ATOS III Triple Scan.

Parameter	Value
Camera pixels (MP)	2 × 8
Measuring volume (mm)	170 × 130 × 130
Measuring distance (mm)	490
Lamp type	LED
Focal length camera lenses (mm)	40
Focal length projector lens (mm)	60
Point distance (mm)	0.055
Reference points (mm)	3
Camera position	SO

**Table 3:** Nominal area of the interface section of each specimen.

Area of Specimen T5 (mm^2^)	Area of Specimen T10 (mm^2^)	Area of Specimen T15 (mm^2^)	Area of Specimen T20 (mm^2^)
6253.03	6250.00	6049.83	6172.62
6116.67	5981.31	6045.55	6224.99
6259.21	6016.51	6075.12	6188.61
6358.86	6084.16	6025.98	6083.29
6379.94	6086.96	6106.65	6334.63

**Table 5:** Volumetric analysis.

Specimen Number	Material Volume (mm^3^)	Defect Volume (mm^3^)	Defect Volume Ratio (%)	Average Pore Diameter (mm)	Maximum Pore Diameter (mm)	Minimum Pore Diameter (mm)
T5	97,751.97	2258.63	2.26	0.820	20.790	0.090
T10	108,022.87	1975.05	1.8	0.540	9.680	0.090
T15	106,446	1872.81	1.73	0.350	7.180	0.100
T20	101,926.78	2456.87	2.35	0.430	10.560	0.090

**Table 6:** Pore analysis of each specimen’s interface area.

Specimen Number	Material Area (mm^2^)	Defect Area (mm^2^)	Defect Area Ratio (%)	Average Pore Diameter (mm)	Maximum Pore Diameter (mm)	Minimum Pore Diameter (mm)
T5	2070.850	34.827	1.68	0.16563	1.253	0.0399
T10	2213.681	51.275	2.31	0.23700	4.039	0.0642
T15	2134.014	47.909	2.245	0.19567	2.038	0.0535
T20	2172.486	15.539	0.715	0.14492	0.493	0.0437
